# Multi-component cognitive intervention for older adults with mixed cognitive levels: implementation and preliminary effectiveness in real-world settings

**DOI:** 10.1186/s12877-021-02489-z

**Published:** 2021-10-12

**Authors:** Hui-Fen Mao, Athena Yi-Jung Tsai, Ling-Hui Chang, I-Lu Tsai

**Affiliations:** 1 School of Occupational Therapy, College of Medicine, National Taiwan University, Taipei, Taiwan; 2 Department of Physical Medicine and Rehabilitation, National Taiwan University Hospital, Taipei, Taiwan; 3 Department of Occupational Therapy/Kaohsiung Medical University, Kaohsiung, Taiwan; 4 Department of Occupational Therapy, College of Medicine/National Cheng Kung University, Tainan, Taiwan; 5 Institute of Allied Health Professions, College of Medicine/National Cheng Kung University, One University Road, Tainan, 701 Taiwan; 6 ZHI XIN Occupational therapy clinic, Yulin, Taiwan

**Keywords:** Cognitive training, Aging, Community-based practice, Implementation fidelity

## Abstract

**Background:**

In most controlled studies of multi-component cognitive intervention, participants’ cognitive levels are homogenous, which is contrary to real-world settings. There is a lack of research studying the implementation of evidence-based cognitive intervention in communities. This study describes the implementation and preliminary effectiveness of a Multi-component Cognitive Intervention using Simulated Everyday Tasks (MCI-SET) for older adults with different cognitive levels in real-world settings.

**Methods:**

Single group, pre-intervention assessment, post-intervention assessment, and 3-month follow-up research design. MCI-SET consists of 12 two-hour weekly sessions that include motor-cognitive tasks, cognitive training, and cognitive rehabilitation. One hundred and thirty participants, > = 65 and frail, dependence on > = one instrumental daily activity, or with confirmed dementia, from eight community centers were included. The primary outcome is general cognition (Montreal Cognitive Assessment-Taiwan, MoCA-T). Secondary outcomes are memory (Miami Prospective Memory Test, Digits Forward, Digits Backward), attention (Color Trail Test-Part 1), executive function (Color Trail Test-Part 2), and general function (Kihon Checklist-Taiwan).

**Results:**

Pre-intervention workshop for group leaders, standardized activity protocols, on-site observation, and ten weekly conferences were conducted to ensure implementation fidelity. MCI-SET had an 85% retention rate and 96% attendance rate. The participants had a mean age of 78.26 ± 7.00 and a mean MoCA-T score of 12.55 ± 7.43. 73% were female.

General cognition (Hedges’ g = 0.31), attention (Hedges’ g = 0.23), and general function (Hedges’ g = 0.31), showed significant post-intervention improvement with small effect size. Follow-ups showed maintained improvement in general cognition (Hedges’ g = 0.33), and delayed effect on attention (Hedges’ g = 0.20), short-term memory (Hedges’ g = 0.38), and executive function (Hedges’ g = 0.40). Regression analysis indicated that the intervention settings (day care centers vs neighborhood centers), the pre-intervention cognitive levels, and the pre-intervention general function of the participants were not associated with the outcomes.

**Conclusions:**

MCI-SET is feasible and can improve the cognitive skills and general functions of older adults with heterogeneous cognitive skills or disabilities. It is essential to tailor programs to fit the interests of the participants and the culture of local communities. Group leaders must also have the skills to adjust the cognitive demands of the tasks to meet the heterogeneous cognitive levels of participants.

**Trial registration:**

This study was retrospectively registered at clinicaltrials.gov (Identifier: NCT04615169).

**Supplementary Information:**

The online version contains supplementary material available at 10.1186/s12877-021-02489-z.

## Introduction

Fifty million people are currently living with dementia and the number is expected to triple to 152 million by 2050 [1]. Since most people with dementia or at a high risk of developing dementia reside in the community, effective community-based programs can help to prevent or manage dementia. Dementia prevention, with the goal of preventing or delaying the development of dementia, targets individuals who have a high risk of developing dementia or are at the preclinical stage where cognitive deficits have not yet led to functional dependence. Dementia management, with the goal of delaying the progression of dementia, targets individuals who have already developed dementia and exhibit clear cognitive decline and functional dependence. There is increasing empirical evidence to support that structured and regular cognitive activities are essential to a brain health lifestyle [2].

Cognitive deficits are typically not the only symptom individuals experience, and these deficits often lead to multiple consequences such as decreased activity levels, difficulties with daily activities, withdrawal from social participation, etc. Cognitive intervention is an increasingly popular approach used to maintain or improve the cognitive function of older adults with or without cognitive impairments [3]. Indeed, appropriate cognitive intervention has the potential to change neuro-mechanisms in the brain [4]. For example, research has shown that the activity in certain areas of the brain in people with mild cognitive impairment increases after training, suggesting that the training improves the brain’s compensatory mechanism and partly restores affected functions [5].

Because of the complexity of the issues that people with cognitive impairments encounter, scholars have advocated for a multi-component intervention to address the different aspects of cognitive problems. Compared to single-component intervention, multi-component cognitive intervention is more effective at improving or maintaining the cognitive functions of older adults [6]. For example, our previous quasi-experimental study included a multi-component cognitive intervention composed of short motor-cognitive dual-task exercises with high cognitive demands, cognitive training with carefully designed cognitive exercises derived from daily activities (e.g., identifying specific items from complex grocery flyers, memorizing the dishes from a table setup, etc.), and cognitive rehabilitation that facilitates use of strategies in daily lives. This intervention improved cognitive skills and functional performance for people with mild cognitive impairments [7].

There is a 8% prevalence of dementia for people over 65 years of age in Taiwan. In addition, Taiwan is predicted to be a super-aged society in 2026. Given these conditions, the Ministry of Health and Welfare in Taiwan published a Dementia Initiative in 2014 to lay out a cohesive dementia prevention and management policy. In 2016, the Ministry of Health and Welfare launched a nation-wide health enhancement project that focused on preventing and delaying the functional deterioration of older adults in communities. The project was to establish a community environment that facilitates a healthy lifestyle by encouraging socialization [8, 9], and by providing a variety of group activities that are evidence-based and health-promoting (such as exercise, nutrition, ADL function, etc.). The Ministry called on universities, health-related professionals, and health-related organizations to submit proposals for evidence-based community activity programs that could be carried out in community centers throughout the nation.

In response to the call, we revised our previous empirically-supported cognitive intervention program [7] to take into account further literature review, clinical experiences, and the guidelines set by the Ministry. The revised protocol, Multiple-component Cognitive Intervention using Simulated Everyday Tasks (MCI-SET), takes 2 hours per session and places more emphasis on tailoring the activities to match the needs of older adults with various cognitive levels. Over 200 proposals were submitted and reviewed by the Ministry, and 78 protocols (including MCI-SET) were approved by an ad hoc expert review committee. The protocols (including the program description, activity protocols, and qualifying professionals who could implement the program) were posted on a governmental website where community centers could choose a program based on the needs of their communities.

It is worth noting that even though there is an abundance of research that supports the efficacy of cognitive intervention, little attention has been paid to investigating the application of evidence-based cognitive intervention in the community, even despite the increasing need for applicable evidence-based research to enable existing community agencies and networks to participate in dementia prevention. Another methodological issue with current evidence is the homogeneity of research participants. To strengthen research designs and standardize treatment protocols, most cognitive intervention research has only included participants with similar cognitive levels, such as those with normal cognition, or those with mild cognitive impairments, or those with dementia, etc. [10]. This homogeneity in participants exists in sharp contrast to what is observed in communities, which are inclusive of people with different cognitive skills. The effectiveness of cognitive intervention with participants of heterogeneous cognitive levels has rarely been examined.

The implementation of MCI-SET offered an opportunity to address these gaps in current knowledge by examining how researchers can use the opportunities afforded by the government and work with professionals and local communities to apply evidence-based intervention for older adults of heterogeneous cognitive levels. We referenced RE-AIM (Reach, Effectiveness, Adoption, Implementation, and Maintenance) framework [11], specifically the aspects of reach, adoption, and implementation, to evaluate the translation and effectiveness of MCI-SET. This paper discusses the feasibility, implementation, and preliminary effectiveness of MCI-SET in eight community settings in two cities in southern Taiwan.

The specific aims of the study are to:

1. Describe the implementation of an evidence-based cognitive intervention (MCI-SET) in community centers.
2. Examine the preliminary effectiveness of MCI-SET to improve general cognition, memory, attention, executive function, and general function.
3. Assess the feasibility of a cognitively-inclusive group approach.

## Methods

The study was conducted from August, 2017 to December 2018. The implementation of MCI-SET is discussed here in terms of intervention development and fidelity monitoring. To evaluate preliminary effectiveness, we used a single group, pre-intervention assessment, post-intervention assessment, and 3-month follow-up research design. The program was provided at multiple community centers (each hosting a single group). The effectiveness of a cognitively-inclusive approach was determined by assessing whether participants’ cognitive levels and general function affected the outcomes at post-intervention, and the feasibility of the program was examined by assessing the required training to execute intervention [11]. This study was approved by National Taiwan University’s Affiliated Hospital’s Ethics Committee for Medical Research and retrospectively registered at clinicaltrials.gov [Identifier: NCT04615169, 04/11/2020]. Informed consent was obtained from all the study participants.

### Participants

Community centers screened and recruited participants who met the criteria set by the Ministry to receive the service. The criteria were: 1) > 65 years old; 2) met the Study of Osteoporotic Fractures (SOF) criteria of frailty [12, 13]; and 3) dependence of at least one instrumental daily activity. People with non-cognitive issues (such as severe visual, hearing, or physical impairments) that affected the completion of evaluations were included in the programs but their data were excluded from the analysis of preliminary effectiveness. Strategies for recruitment included flyers, partnerships with other neighborhood organizations, word-of-mouth, etc. Strategies to maintain attendance included reminders sent via text messages and community broadcast systems, assistance with transport, use of concurrent activities as incentives (for example, provision of a communal lunch after the session), etc.

One hundred and fifty-eight participants completed pre-tests and 28 were dropped from the program due to the following reasons: declined to complete the post-test (1 person) or were absent for more than five sessions (27 persons). There was no significant difference found in age, gender, and MoCA scores between those that dropped out and those that were included in the analysis (all *P* > 0.05). The reported reasons for participants completing less than 10 sessions or missing the follow-up test were scheduling conflicts and occasional inconveniences, such as heavy rain and transportation issues.

### Study intervention

MCI-SET is a 12-session, 2-hour weekly group multi-component cognitive intervention that included motor-cognitive exercise, cognitive training, and cognitive rehabilitation. We also take into consideration the practicality of conducting a group intervention in a community center, such as equipment needed, space, and adherence. The activities used in cognitive training were contextually-relevant simulated everyday cognitive tasks. We developed 24 standardized activity protocols with an emphasis on tailoring the cognitive activities to match the needs of older adults with various cognitive levels. Opportunities for social interaction were deliberately incorporated into the intervention, as familiarity among participants improves attendance and increases their motivation to engage in the programs. Therapists were also encouraged to communicate with the administration about the goals and content of the intervention (see supplementary file for more detailed descriptions of the intervention structure).

### Outcome measures

The participants were assessed in the first week of the 12-week intervention, reassessed within a week of completing the intervention, and followed up after 3 months. The activity patterns and care of the participants between post-intervention and follow-up were similar to pre-intervention. Occupational therapists trained in test administration completed the assessments.

This study adopted general cognition as the main outcome. Memory, attention, executive function, and general function was the secondary outcomes to explore the effects of MCI-SET. General cognition was measured by the Montreal Cognitive Assessment-Taiwan (MoCA-T) [14, 15]. The maximum score of MoCA-T is 30, with higher scores indicating better cognition. The total score of MoCA-T is adjusted for educational effects. A cut-off score of 21/22 can differentiate persons with normal cognition or dementia with a 98% sensitivity and a 95% specificity [15].

Functional episodic memory was assessed using the event-based task in the Miami Prospective Memory Test (MPMT) [16]. Participants were asked to pick up an envelope on a desk, obtain a fixed amount of paper bills from the envelope, give a bill to the examiner and put another in a different envelope within 30 min. They were scored 0–9 based on “intention to perform, accuracy, and need for reminders,” with higher scores indicating a better performance.

WAIS-IV Digit Span subtest (Digits Forward, DF, and Digits Backwards, DB) measures short-term memory (DF) and working memory (DB). Participants listened to a sequence of digits and were asked to recall it in order (DF) or in reverse-order (DB). Total scores ranged 2–9 for DF and 2–8 for DB [17], with higher scores indicating better performance.

Attention was measured by the Color Trail Test Part 1 (CTT-1) and executive function was measured by Part 2 (CTT-2) [18]. With CTT-1, participants were asked to quickly connect 25 numbered circles. With CTT-2, participants were asked to quickly connect 25 numbered circles while alternating between pink and yellow colors (1-pink, 2-yellow, 3-pink, etc.). Shorter times indicated better performance.

General function was measured by the Kihon Checklist-Taiwan (KC-T) [19, 20]. KC-T is used by the Ministry of Health and Welfare as an outcome indicator for community-based programs that delay and prevent disability [9]. KC-T is a self-reported questionnaire, consisting of 25 items divided into 7 sub-categories: general independence, physical strength, nutrition, oral function, level of social activities outside the home, cognitive function, and risk of depression. Each item is rated as pass (0) or fail (1) so a higher total score indicates a lower level of function [19].

### Statistical analysis

Demographic characteristics and index results were analyzed using descriptive statistics. Continuous variables were reported with mean ± standard deviation (SD). One-way repeated measurement ANOVA test was used to check the overall significance between pre-tests, post-tests, and follow-ups. For the comparisons between two dependent variables, Student’s paired t-test or Wilcoxon signed-rank was used based on the normality assumption. For multiple comparisons, Bonferroni was used to control the inflation of type I error (alpha). The results were further analyzed by calculating the effect sizes (Hedges’ g), defining 0.2 as small, 0.5 as medium, and 0.8 as large [21]. Linear regression analysis was used to examine whether individual characteristics (age, gender, education, general cognition, and general function at baseline) and organizational effects (day care and neighborhood centers) could significantly predict intervention benefits. Significance was set at *p* < 0.05. All analyses were performed using IBM SPSS 20 [IBM Corp., Somers, New York, USA].

## Results

### Aim 1: implementation of the MCI-SET program in the community

In terms of number of downloads, MCI-SET ranked number 5 in the Ministry-approved community activity programs as of September 2020, showing that MCI-SET received high attention among all the approved protocols. To assess the implementation and preliminary effectiveness of MCI-SET, eight community centers (4 day care centers for older adults with disabilities and four neighborhood centers) were recruited to participate in this study.

One hundred and fifty-eight participants completed pretests and twenty-eight dropped out of the program, indicating an 85% retention rate. 96% of the remaining participants attended 10–12 of the 12 sessions. The positive responses of participants and community organizers supported the utility of disseminating and implementing MCI-SET. A staff member reported, “It was really good to have professionals coming to organize and lead the intervention. Mr. A [a person with mild to moderate dementia] made significant progress [after attending the session] … Instead of sitting and waiting, he started to help with placing things in the refrigerator and setting the table [for meals].”

### Aim 2: preliminary effectiveness of MCI-SET in real-world settings

One hundred and thirty participants who completed all three assessments were analyzed to examine the preliminary effectiveness of MCI-SET. The participants had a mean age of 78.26 ± 7.00, a mean of 5.85 ± 4.10 years of education, and 73% were female.

The participants showed significant post-intervention improvement in most outcomes: general function (KC-T), general cognition (MoCA-T), and attention (CTT-1) with small effect. Follow-ups showed that improvement in general cognition and attention remained significant with small effect. The changes in short-term memory (DF) and executive function (CTT-2) were not significant immediately after the intervention but showed significant improvements at follow-ups. Episodic memory (MPMT) and working memory (DB) showed no significant changes after intervention (Table 1).

**Table 1:** Comparison of pre-test, post-test, and 3-month follow-up *(n* = 130) and the effect size (Hedges’ g)

Outcomes	Pre-test	Post-test,	Follow up,	Pre - Post Changes,	*P*-values	Hedges’ g	Pre - Follow Changes,	*P*-values	Hedges’ g
	M (SD)	M (SD)	M (SD)	M (SD)			M (SD)		
KCT	10.17 ± 4.07	9.14 ± 4.05	9.56 ± 4.71	−1.03 ± 2.75	< 0.001*	0.37	0 ± 0	1.000	0
MOCA (general cognition)	12.55 ± 7.43	14.11 ± 8.54	14.05 ± 8.16	1.56 ± 5.12	< 0.001*	0.31	1.13 ± 3.41	< 0.001*	0.33
MPMT	3.36 ± 3.02	3.42 ± 3.10	4.15 ± 3.44	0.05 ± 2.16	0.751	0.03	0.58 ± 3.15	0.040	0.18
Digits Forward	6.04 ± 1.68	6.26 ± 1.50	6.46 ± 1.43	0.22 ± 1.35	0.052	0.16	0.47 ± 1.24	< 0.001*	0.38
Digits Backward	2.80 ± 1.44	2.89 ± 1.57	2.92 ± 1.41	0.13 ± 1.05	0.285	0.12	0.05 ± 1.04	0.701	0.05
CTT1	188.59 ± 108.69	170.01 ± 108.64	170.75 ± 113.65	−14.41 ± 63.41	< 0.001*	0.23	−15.09 ± 74.73	< 0.001*	0.20
CTT2	267.37 ± 116.84	257.04 ± 135.68	224.68 ± 99.58	−9.97 ± 104.95	0.020	0.10	−24.02 ± 60.26	0.002*	0.40

*Note*. Kihon Checklist-Taiwan (KCT), *MOCA* Montreal Cognitive Assessment, *MPMT* Miami Prospective Memory Test, *CTT-1* Color Trail Test-Part 1, *CTT-2* Color Trail Test-Part 2

*significant level *P* < 0.0036. There were 14 paired tests, after Bonferroni correction, the corrected alpha is0.0036 (0.05/14)

Regression analysis results show that pre-intervention cognition levels, general function, and the different settings of the program (day care centers vs neighborhood centers) were not associated with the improvement of general cognition and general function. Instead, high attendance was significantly associated with improving general function (Table 2).

**Table 2:** Univariate linear regression results of pre-post improvement

Parameters	Change of MoCA		Change of KCT	
	B (95% CI)	P	B (95% CI)	P
Age	−0.05 (− 0.18 to 0.08)	0.414	0.06 (− 0.02 to 0.14)	0.118
Gender				
man	ref.	–	ref.	–
woman	−0.52 (−2.53 to 1.49)	0.608	−0.73 (− 1.91 to 0.44)	0.218
Years of education	−0.13 (− 0.35 to 0.09)	0.254	− 0.06 (− 0.18 to 0.07)	0.375
Attendance	0.15 (− 0.72 to 1.03)	0.731	−0.86 (− 1.59 to − 0.14)	0.020^*^
Organization				
Neighborhood centers	ref.	–	ref.	–
Day care centers	−1.25 (−3.10 to 0.59)	0.181	0.54 (−0.50 to 1.59)	0.305
Pre_KCT	0.01 (−0.24 to 0.26)	0.958	–	–
Pre_MOCA	–	–	−0.04 (− 0.11 to 0.03)	0.279

*Note. KCT* Kihon Checklist-Taiwan, *MOCA* Montreal Cognitive Assessment

### Aim 3: assess the feasibility of a cognitively-inclusive group design

Feasibility was assessed through the training needed to execute the program, the mechanism of monitoring treatment fidelity, and the effects of contextual factors, such as organizational characteristics, on the effectiveness of the program [11]. Eighteen therapists were recruited to implement MCI-SET at the eight community centers. Pre-intervention training focused on equipping the therapists with the knowledge and skills required to implement MCI-SET and included six hours of lectures on theoretical frameworks, assessments, and current evidence of cognitive intervention, as well as six hours of workshops on cognitive activity analysis and modification and grading of the activities according to cognitive demands. Twenty-four standardized activity protocols were also designed for the intervention. Special attention was directed towards how to tailor the cognitive demands of the intervention tasks to match the individual cognitive skills of the participants.

During the three-month intervention period, the therapists attended ten weekly online conferences led by the LHC and FHM to share and discuss their implementation experiences; topics included how to make adjustments to the activities to better fit the different cognitive abilities of participants, what strategies could increase or decrease the cognitive demands of an activity in a group setting, and what responses they observed of the participants. We also went to the community centers to observe the sessions in order to understand and supervise the fidelity of the implementation.

## Discussion

This study describes the implementation of the evidence-driven and pragmatic Multiple-component Cognitive Intervention using Simulated Everyday Tasks (MCI-SET), and examines its feasibility and preliminary effectiveness. The results show that it is feasible to carry out MCI-SET for a group of older adults with heterogeneous cognitive levels. The program had a high attendance and follow-up rates. The findings also support preliminary effectiveness in improving general cognition and attention of the participants both immediately post-intervention and at follow-ups; while general function showed improvement immediately post-intervention, but did not show improvement at follow-ups. There was also a delayed improvement in memory and executive function (CTT-2) at follow-ups. The effect size of MCI-SET was comparable to the results of a recent meta-analysis indicating the cognitive training effects for persons with MCI on executive function (Hedges’ g [0.95 CI]: 0.29 [0.16–0.43]) and short-term memory (Hedges’ g [0.95 CI]: 0.334 [0.10–0.56]) [22].

MCI-SET was developed in a previous study for persons with mild cognitive impairments [7]. The results of the current study now support the applicability of MCI-SET to persons with or without dementia. Approximately 81% of the participants in this study had MoCA-T scores lower than 21; that is, they had cognitive levels similar to those with dementia [15]. Most studies suggest that cognitive training has limited benefits for persons with more advanced cognitive impairments, although high-intensity training for a few hours a day is a notable exception [10]. A possible explanation for the progress demonstrated in this study could be that all the participants received adequate cognitive stimulation to bring about the changes. Strategies were used to keep the participants cognitively engaged throughout the sessions. First, the occupational therapists were trained to grade the cognitive demands of the therapeutic tasks according to the responses of individual participants. To prevent participants from quitting when tasks are too easy or difficult, as often observed in older adults with cognitive impairments, the difficulty of the tasks was closely monitored and adjusted as needed so that the participants were able to maintain engagement. Second, the intervention was conducted in a group setting where opportunities for interaction among the participants were deliberately integrated, which contributed to the continual engagement of the participants during the 2-hour sessions.

Regression analysis also shows that the independent variables in this study were not significantly associated with the outcomes of general cognition and general function and —all participants showed improvement, regardless of their cognitive skills. In comparison to most studies, which include rigorously controlled research designs with participants of homogenous cognitive levels, neighborhood health-promoting programs are more focused on increasing participation and creating an environment that enables older adults to remain in the community as their cognitive skills change, thus necessitating the inclusivity of participants with heterogeneous cognitive levels. This study supports the feasibility and effectiveness of a cognitively inclusive group design for persons with heterogeneous cognitive skills.

As indicated by the significant changes in KC-T score, our study adds to the emerging evidence that cognitive intervention is also beneficial to general function and can potentially reduce disability risks [23, 24]. This progress can be also attributed to the inclusion of physical exercises and the discussions on cognitive problems encountered in participants’ daily lives; both have been shown to be potentially effective in reducing disability [25]. Attending the weekly intervention also increased participants’ activity levels and social engagement, which has been shown to be effective in improving physical function [26]. However, psychological assessments (such as depression or quality of life) were not included in this study, and can be explored in future research.

This study examined several aspects of implementing MCI-SET: reachability, adoption by the community in terms of recruitment and maintenance strategies, effects of organizational characteristics, training required to execute the program, and implementation fidelity [11]. Reachability examines whether the program can be delivered to the targeted population by determining whether the participants actually represent the targeted population [11, 27]. Persons of older age and lower education, who are most at risk of dementia, are at the frontline of dementia prevention [28]. Participants in this study were an average of 78.6 years old and had only received education for an average of 5.84 years. This shows that MCI-SET did reach the intended targeted population.

Although organizational characteristics have rarely been evaluated in the implementation of cognitive intervention, researchers have begun to recognize how organizational features can influence the delivery of health promotional programs [29]. In this study, MCI-SET was delivered in day care centers for older adults with disabilities or in neighborhood centers. During field observations and online conferences, which were conducted to ascertain implementation fidelity, it was revealed that the dynamics at day care centers and neighborhood centers were very much in flux and posed potential challenges to implementation fidelity. Neighborhood centers were open to the public and often lacked an enclosed activity room similar to those in day care centers. The size of the groups often fluctuated because local residents requested to join the program impromptu, mid-session, and/or some participants had to leave early. The length of the sessions sometimes needed to be adjusted at the request of other community-event organizers.

We had anticipated that these disruptions in neighborhood centers would negatively impact the effectiveness of the program. However, the results show that the improvements in general cognition and general function were not associated with whether the program was delivered in day care centers or in neighborhood centers. It could be that the therapists were able to respond to community needs without compromising the treatment fidelity because of our fidelity mechanism, which included pre-intervention workshops and weekly online conferences, the latter focusing on sharing experiences and discussing strategies and intervention protocols. Although we had implemented several qualitative strategies to ensure intervention fidelity, a checklist of fidelity indicators would have been helpful.

There are some limitations to this study. First, the recruitment was based on convenient sampling. It was up to community organizers to select MCI-SET and participation in the program itself was voluntary. Participants’ motivation may have also influenced the results. We were unable to obtain information regarding participants’ dementia diagnosis and medications, and thus cannot rule out the influence of antidepressant or anti-dementia medication on the intervention effect. However, these limitations are reflective of real-world situations where older adults usually attend health-promoting programs voluntarily and the community centers often avoid asking the participants about mental health history. Second, this study does not have a control group because this study was conducted following the directive of a nation-wide health enhancement project and limited by the resources provided by the government. Third, MCI-SET continued to have an effect even after intervention. We are unable to examine the mechanism of these effects and strategies (such as booster sessions) to strengthen the effects due to resource limitations, but this can be explored by future research. Lastly, MCI-SET requires a trained professional to deliver the program. While this helps ensure the quality of the intervention, the need for such a trained professional may limit widespread implementation because community centers might not have funding or qualified occupational therapists available.

## Conclusion

In most controlled studies of multi-component cognitive intervention, participants’ cognitive levels are homogenous. There is a lack of research studying the actual implementation of evidence-based cognitive intervention in communities. This study has provided evidence supporting the feasibility and preliminary effectiveness of the evidence-based and pragmatic Multi-component Cognitive Intervention using Simulated Everyday Activities (MCI-SET). The intervention is highly accepted in communities and improves the general cognition, memory, attention, executive function, and general function of older adults with different cognitive levels.

This study provides evidence to mend the theoretical “leaky pipeline” of intervention for research purposes to execution of empirically and theoretically robust interventions in communities [30]. Future research can examine essential elements of the MCI-SET that can be further structured and developed into a fidelity checklist for widespread implementation.

## Supplementary Information

**Additional file 1.**

## Data Availability

The database used and analyzed during the current study are available from the corresponding author on reasonable request.

## Abbreviations

CTT-1: Color Trail Test-Part 1
CTT-2: Color Trail Test-Part 2
DF: Digits Forward
DB: Digits Backwards
KC-T: Kihon Checklist-Taiwan
MCI-SET: Multi-component Cognitive Intervention using Simulated Everyday Tasks
MPMT: Miami Prospective Memory Test
MoCA-T: Montreal Cognitive Assessment-Taiwan version
