# Supplementary material for: Multi-component cognitive intervention for older adults with mixed cognitive levels: implementation and preliminary effectiveness in real-world settings
Source: BMC Geriatr. 2021 Oct 12;21:543. doi: 10.1186/s12877-021-02489-z (PMC8507169; doi:10.1186/s12877-021-02489-z)
Supplement: Supplementary file 1 — Additional file 1. [file 12877_2021_2489_MOESM1_ESM.docx]

Here are the key findings from the literature, and from our experiences of health promotion in the community, that informed the development of the Multi-component Cognitive Intervention using Simulated Everyday Tasks (MCI-SET) intervention:

1. Extensive empirical evidence supports that cognitive training maintains or improves cognitive skills, and that cognitive rehabilitation improves the cognitive functional performance of older adults with or without cognitive impairments.
2. Recent advances in research show that motor-cognitive dual-task exercises improve cognition (Harod, Hamacher, Schega, & Muller, 2018; Netz, 2019).
3. There may be issues regarding technological resources in communities, such as a frequent lack of funding for computers and for the purchase of cognitive exercise computer programs that are commonly used in cognitive intervention.
4. Physical exercises are important and highly acceptable to older adults.
5. Cognitive exercises similar to everyday tasks are often more interesting to older adults than decontextualized cognitive exercises conducted with paper and pencil.
6. Participants in cognitive groups in community settings would be composed of older adults with heterogenous cognitive levels. Therefore, it is important to have activities that can be adjusted on the spot in order to best fit the cognitive abilities of the participants.
7. Opportunities for social interaction should be incorporated carefully into the interventions, as familiarity among participants improves attendance and increases their motivation to engage in the programs.

MCI-SET session consists of:

(1) Warm-up: 15 to 30 minutes of motor-cognitive exercises that arouse consciousness and awareness and provide cognitive stimulation. For example, the participants may sing a song, mimic the dance movements of the group leader, or clap their hands when specific words occur in the song.

(2) Two 25-minute repetitions of contextually-relevant simulated everyday cognitive tasks that are guided by the group leader, with a 15 to 20-minute break in between. Each session is led by a group leader and another co-leader. The group leaders adjust the cognitive demands of the activities for the group as a whole or for individual participants to create a learning environment centered around reducing errors (Dechamps et al., 2011).

Cognitive tasks address complex attention, visual scanning, auditory attention, visual and motor memory, auditory memory, prospective memory, executive function, problem solving, etc. (see Chang et al., 2020 for details of the intervention protocols). In contrast to the decontextualized tasks conducted with paper and pencil used in most traditional cognitive training tasks, the activities here require real-life skills and use everyday tools. The activities are carefully designed to reflect local cultures and the cognitive difficulties that people with cognitive impairments experience in their daily lives (Wen, Mao, Chang, & Chiu, 2016).

For example, during the week focused on visual attention and memory training, the participants are given a series of pictures featuring traditional food-offerings. The participants are asked to find a specific food, which requires visual-scanning skills, or to memorize the types of food in the dish, which requires memorization skills. The group leader modifies the activities according to the responses of the participants to adjust the cognitive skills required. For example, increasing the complexity of the dishes or reducing the time allotted to finish the task so as to challenge their cognitive abilities. The group leader may also turn the cognitive task into a competitive game by dividing the group into small groups to make the task more fun and engaging.

During a session, the difficulty of tasks is gradually increased as the participants become familiar with the tasks. Whenever possible, assistance (such as cues) given to each participant is adjusted to best fit the task demands and their individual skill level.

3) 20-25 minutes of participant-centered group discussions that focus on daily problems associated with cognitive impairments and applications of cognitive strategies in everyday lives. Specifically, participants identify real-life tasks that require similar cognitive skills and strategies used in the cognitive training and discuss how to apply the skills and strategies to their daily lives.

References:

Dechamps, A., Fasotti, L., Jungheim, J., Leone, E., Dood, E., Allioux, A., . . . Kessels, R. P. (2011). Effects of different learning methods for instrumental activities of daily living in patients with Alzheimer's dementia: a pilot study. *American Journal of Alzheimers Disease and Other Dementia, 26*, 273-281. doi: 10.1177/1533317511404394

Harod, F. Hamacher, Schega L. & Muller, N. G. (2018). Thinking while moving or moving whilethinking: Concepts of motor-cognitive training for cognitive performance enhancement. Frontiers in Aging Neuroscience, 10, 228-239. https://doi.org/10.3389/fnagi.2018.00228.

Netz Y. (2019). Is there a preferred mode of exercise for cognition enhancement in older age?: A narrative review. *Frontiers in medicine*, *6*, 57-67. https://doi.org/10.3389/fmed.2019.00057

Wen, M. H., Mao, H. F., Chang, L. H., Chiu, M. J. (2016). A qualitative study of the instrumental activities of daily living for mild cognitive impairment and caregivers. Paper presented at The American Occupational Therapy Association, Chicago, USA.
